# The *Bitter Taste Receptor (T2R)* Gene Repertoire in the Porcine Circumvallate Papillae Consists of Fourteen Genes, Including Two Newly Validated *T2R61* and *T2R62*

**DOI:** 10.3390/genes17040400

**Published:** 2026-03-31

**Authors:** Xinle Tan, Kar Wai Lai, Shuyu Yang, Miaomiao Zhou, Maik Behrens, Eugeni Roura

**Affiliations:** 1 Nutrition and Chemosensory Science, Centre for Animal Science, Queensland Alliance for Agriculture and Food Innovation, The University of Queensland, Brisbane, QLD 4072, Australiakarwai.lai@uq.edu.au (K.W.L.); shuyu.yang@uq.edu.au (S.Y.); miaomiao.zhou1@student.uq.edu.au (M.Z.); 2 School of Food Science and Engineering, South China University of Technology, Guangzhou 510641, China; 3 Leibniz Institute for Food Systems Biology, Technical University of Munich, 85354 Freising, Germany; m.behrens.leibniz-lsb@tum.de

**Keywords:** bitter taste receptors, circumvallate papillae, pig transcriptome, taste perception

## Abstract

Background/Objectives: Bitter taste perception is important for pig feeding behavior and survival. The type 2 taste receptors (T2Rs) are G protein-coupled receptors responsible for the sense of bitter taste perception in mammals. *T2Rs* are expressed in taste receptor cells located in the taste buds of the papillae of the tongue and in other tissues such as the gastrointestinal tract, respiratory epithelia, and immune system. In pigs, twelve *T2R* genes have previously been experimentally identified, although only a limited number of studies have investigated this gene family. We hypothesized that the full *T2R* gene repertoire in pigs has yet to be uncovered. Methods: Circumvallate papillae (CVP) were collected from 12 pigs, and a combination of bioinformatics analysis and experimental validation was used to identify and annotate *T2R* transcripts in the pig transcriptome. The CVP transcriptome was explored using reference-guided assembly to identify potential novel transcripts, and newly identified protein-coding transcripts were confirmed by PCR and Sanger sequencing. Results: The results confirmed significant expression of 10 of the 12 known *T2Rs (3*, *4*, *7*, *9*, *10*, *16*, *20*, *39*, *41*, and *60*). Two novel *T2R* transcripts (ENSSSCT00000089410.2 and ENSSSCT00000091318.1) were discovered and referred to as *T2R61* and *T2R62*. *T2R62* contained larger exons than those annotated in the reference genome. The results also showed that porcine *T2R20* is a member of the porcine *T2R* family highly similar to several human *TAS2Rs*, including *TAS2R20* (*TAS2R49*). In total, the porcine *T2R* repository contains 14 transcripts supported by strong evidence. Conclusions: This study expands knowledge of the porcine *T2R* repertoire and provides insight into the genetic basis of taste perception, food selection, nutrition, and adaptation biology in pigs.

## 1. Introduction

Bitter taste perception may have evolved as a defense mechanism against the ingestion of potential toxic compounds [1,2]. It plays an essential role in determining feeding choices and ultimately survival in many animal species, including pigs [3,4,5]. The *type 2 taste receptor* (*T2R*) genes encode bitter taste receptors that recognize a wide range of chemical compounds in food [4]. In the oral cavity, *T2Rs* are expressed mainly in the taste sensory cells forming the taste buds, which, in turn, form clusters contained in one of the three types of tongue papillae: the fungiform, the foliate or the circumvallate [6]. They are also characterized in other extra-oral tissues, such as the gastrointestinal tract in mammals, including pigs [7,8]. Gut T2Rs were related to hormone regulation and gastrointestinal mobility [9,10]. The size of the T2R repertoire is species-specific [11,12,13]. There are ~26 *T2Rs* in humans [14,15], 35 in mice [16], 12 in cats [17] and three in chicken [18], to name just a few examples. In pigs, the *T2R* gene family has been identified and characterized [4,8]. Recent papers have reported expression of 12 *T2R* genes, mostly based on experimental evidence [4,19]. A search in Uniprot (a protein database) using the key words “T2R sus scrofa” returned 101 records, many being associated to duplicated gene names and lacking experimental support at protein level. Thus, the pig *T2R* receptor repertoire needs to be updated with more experimental evidence. The characterization of the full functional *T2R* repertoire in pigs is fundamental for improving feeding practices related to alternative feed ingredients, efficiency of feed use, impact on gut health and optimization of pig nutrition practices in general [20]. In this study, we hypothesize that the repertoire of *T2R* genes in pigs has not yet been fully characterized. Thus, it was envisaged that a combination of bioinformatic tools together with experimental validation would lead to the identification and verification of unconfirmed *T2R* transcripts (and genes) in the pig. Circumvallate papillae were chosen as the study tissue because they have a high concentration of taste buds and sensory cells. Additionally, their large size and clear visibility make them easy to dissect, minimizing the inclusion of non-taste tissue. Moreover, given the growing interest in how taste receptor genes may relate to production traits in pigs, understanding the full *T2R* repertoire also provides an essential foundation for future association studies investigating potential links between *T2R* variation and phenotypic outcomes.

## 2. Materials and Methods

### 2.1. Animal Ethics

This research was approved by the University of Queensland Animal Research Ethics Committee (Approval No. CNFS/QAAFI/192/20/HMRC).

### 2.2. Animals and Sampling

This study was designed as a descriptive, exploratory investigation to identify novel transcripts in the circumvallate papillae (CVP) of healthy Large White pigs. No specific treatments were assessed, as the primary objective was to characterize baseline gene expression in healthy tissue. Twelve Large White piglets from different litters (25 days of age, weighing 7.8 ± 0.6 kg) were individually housed in rooms G2 and G3 of the University of Queensland Herston Medical Research Centre (Brisbane, QLD, Australia). The rooms consisted of 6 slatted-floor pens (1.64 sqm each) and are equipped with an environmental control system. The temperature in the nursery room where the study was conducted was set at 30 °C, and humidity and temperature were monitored regularly. The pigs were exposed to 10 h of light per day, programmed from 7.00 h to 17.00 h, with a light intensity between 40 and 60 lux. The pigs were monitored daily for any signs of health issues, and any symptoms were reported by animal carers, including diarrhea, heavy breathing/coughing, dehydration, weight loss, furry skin, pale mucosa, swollen joints, severe scratches, lesions, or eye infections. Each pen was equipped with a nipple drinker and a feeder with multiple feeding spaces, allowing ad libitum access to water, which were checked daily throughout the study to ensure an adequate supply of water and fresh feed.

At the end of the first two weeks of adaptation, the circumvallate papillae are well-developed. The piglets (13.1 ± 1.7 kg) were euthanized by carbon dioxide (CO_2_) inhalation. The CVP were identified based on their distinct gross anatomy as the large, circular structures surrounded by a prominent trench, located bilaterally at the posterior base of the tongue. Using sterile fine forceps and dissecting scissors, each CVP was carefully lifted, and the connective tissue at its base within the trench was cut to harvest the entire papilla. CVP were carefully removed with a scalpel from pig tongue, snap frozen in liquid nitrogen and stored at −80 °C until further use. This method ensures the inclusion of the taste bud-containing epithelium and the underlying lamina propria while minimizing contamination from the surrounding non-gustatory lingual epithelium.

### 2.3. Taste Bud Transcriptome Extraction and Sequencing

Total RNA was isolated from CVP using RNeasy Kits (Qiagen, Venlo, The Netherlands), including a DNase digestion step to avoid genomic DNA contamination, as per the manufacturer’s instructions. Samples were processed in a randomized order for RNA extraction and library preparation to minimize potential batch effects. The quality and quantity of the RNA samples were examined using a NanoDrop™ spectrophotometer (ThermoFisher Scientific, Waltham, MA, USA) and Agilent 2100 Bioanalyzer (Agilent Technologies, Santa Clara, CA, USA). RNA samples with an Integrity Number ≥  8, a 260/280 ratio between 2 and 2.1, and a 260/230 ratio between 2 and 2.2 were used for sequencing. The 12 pig poly(A) RNA samples were sequenced on an Illumina NovaSeq platform (Illumina, San Diego, CA, USA) for a 100 bp single end run. The polyA enrichment was conducted with the Illumina Stranded mRNA workflow (Illumina, San Diego, CA, USA) according to the manufacturer’s instructions. The resulting data were archived in Sequence Read Archive (SRA) in the National Center for Biotechnology Information (NCBI) with BioProject ID: PRJNA1153759.

### 2.4. Transcript Quantitation

The sequencing result was demultiplexed, and quality control was applied using FastQC. Low-quality reads were removed by trimmomatic [21] (Galaxy Version 0.36.6), and high-quality reads were mapped against the pig reference genome (Sus_scrofa.Scrofa11.1.108) using HISAT2 [22]. The criteria for removing low-quality reads are: SLIDINGWINDOW:4:20, LEADING:3, TRAILING:3, MINLEN:36. The aligned reads were summarized by featureCounts [23] (Galaxy Version 2.0.3+galaxy1) to generate a normalized count number for downstream quality control analysis. DESeq2 normalization was applied here due to its ability to account for variability in sequencing depth and biological variability, providing more accurate and reliable results [24].

Transcript assembly was guided with StringTie [25] (Galaxy Version 2.2.1+galaxy1) and merged by StringTie merge (Galaxy Version 2.2.1+galaxy1) to create a non-redundant transcript list following HISAT2 mapping as above. The obtained list was annotated with GFFcompare using the suscrofa11.1 annotation (from ENSEMBL transcript database, Sus_scrofa.Scrofa11.1.108). The nucleotide sequences of all transcripts were extracted with GFFread [26] and further translated to amino acid sequences by Augustus [27] (Galaxy Version 3.4.0+galaxy1). The obtained amino acid sequences were used for a database search using the BLAST algorithm (version 2.17.0) against pig proteome (UP000008227) and human proteome (UP000005640) for homology analysis to identify any potential proteins with similarity to existing T2Rs in pig and human. The blast search was applied using the NCBI blastp command line tool, with an e-value cutoff of 1 × 10^−30^ being considered similar. The experimentally identified pig transcripts with high similarity (e-value < 1 × 10^−30^) to existing human and pig *T2Rs* were further checked with PCR and Sanger sequencing. Protein structures of T2Rs other than a newly identified *T2R* (named *T2R62*) were predicted by AlphaFold and retrieved from the AlphaFold Protein Structure database (https://alphafold.ebi.ac.uk/ (accessed on 16 March 2023)). The T2R62 protein structure was predicted by AlphaFold Colab [28]. The PyMol (Molecular Graphics System, Version 2.0 Schrödinger, LLC, New York, NY, USA) program was used for structure similarity visualization.

### 2.5. Principal Component Analysis (PCA)

To assess global transcriptomic similarities and identify drivers of variation, principal component analysis (PCA) was performed using RStudio (Version 2022.12.0+353). Prior to analysis, raw count data were normalized and transformed via Variance Stabilizing Transformation (VST) within the DESeq2 package (Version 3.19) to account for sequencing depth and biological variability. PCA coordinates and feature loadings were extracted using the prcomp function, which employs Singular Value Decomposition (SVD) to calculate the eigenvectors of the covariance matrix. Final 2D score plots and loading biplots were visualized and formatted using GraphPad Prism 10.

### 2.6. PCR Identification of Transcripts

Our analysis specifically targeted the verification of computationally predicted *T2R* genes that lacked experimental transcript evidence in public databases. The purpose of PCR on the transcripts encoding novel T2Rs was to verify their expression in vivo. Therefore, a series of primers was designed using OligoPerfect Primer Designer (ThermoFisher, USA). The expression of the transcripts was confirmed by PCR using PCR Enzymes & Kits (Qiagen, Venlo, The Netherlands). The primers used in this study are listed in Table 1. The products were subjected to agarose gel electrophoresis on 1.5% agarose gels. The samples with amplicons of the expected sizes were sent for Sanger sequencing to Genetic Research Services (The University of Queensland, Brisbane, QLD, Australia).

### 2.7. Phylogenetic Analysis of T2Rs

The *T2R* amino acid sequences were submitted for alignment and phylogenetic analysis by MUSCLE (MUltiple Sequence Comparison by Log- Expectation, https://www.ebi.ac.uk/Tools/msa/muscle/ (accessed on 16 March 2023) using the default setting and output in ClustalW format. The phylogenetic tree was built by Maximum Likelihood with bootstrap resampling for 1000 times using IQ-TREE [29]. The resulting trees were visualized by iTOL (https://itol.embl.de/ (accessed on 16 March 2023)).

## 3. Results

### 3.1. Characterization of Novel T2R Transcripts

**Figure 1:**
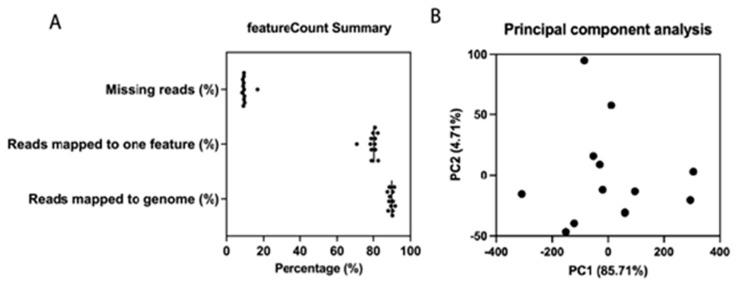
(**A**) featureCount summary showing the percentage of reads mapped to the genome and features or missing reads. (**B**) Principal component analysis of the transcriptomics data using normalized counts by DESeq2. Dots represent different biological samples.

In our study, we utilized high-throughput RNA sequencing (RNA-seq) with an average sequencing depth of 37,277,444 reads and 97.00% mapping efficiency, ensuring comprehensive coverage of the pig transcriptome. The data were processed using reference-guided assembly, with tools such as HISAT2 for alignment and StringTie for transcript assembly. This approach allows for accurate detection and quantification of both known and novel transcripts [30]. FastQC showed that sample quality was adequate for subsequent analysis with >91% bases above Q30 across all samples. The sample reads were then aligned to the reference genome, annotated and summarized by featureCount. The summary, as in Figure 1A, shows that on average, 89.65% reads were mapped to the genome, 79.55% of the reads were mapped to a single feature, and 9.86% of the reads were mapped to the genome but not to any known features (missing reads). From the 12 samples, there were 58,422 transcripts annotated successfully by GffCompare and 30,587 transcripts assembled but not annotated. Normalized counts from each transcript were then used to conduct principal component analysis (PCA), as shown in Figure 1B. Principal Component 1 (PC1) explains 85.71% of the total variance, while PC2 explains only 4.71%. In total, PC1 and PC2 have explained 92.42% of the total variance. This large number is consistent with the fact that the pig samples were under the same biological (including genetic background) and environmental conditions.

Our analysis detected numerous taste signaling elements, including genes associated with taste bud cell markers and signaling pathways, for example: ENSSSCT00000003753 encodes for *T1R1*, ENSSSCT00000060794 encodes for *T1R2*, and ENSSSCT00000047431 encodes for *T1R3*. They are all umami and sweet receptors [31]. ENSSSCT00000009551 encodes for *otopetrin 1*, ENSSSCT00000025573 encodes for *otopetrin 2*, and ENSSSCT00000018745 encodes for *otopetrin 3*. They are all proton-selective ion channels expressed in many different tissues, including taste receptor cells [32].

The amino acid sequences from all the identified transcripts were subjected to NCBI blastp analysis against previously identified T2Rs in pig and human proteomes. An e-value < 1 × 10^−29^ was considered similar to existing annotated proteins. Amongst the hits, 13 of the encoded proteins were identified as similar to human or porcine known T2Rs, as shown in Table 2. Our results also showed that 10 of the known pig *T2Rs* (numbers *3*, *4*, *7*, *9*, *10*, *16*, *20*, *39*, *41*, and *60*) were identified by the RNAseq analysis, which is consistent with previous literature [4]. Other previously reported *T2Rs* were not confirmed by our gene expression analysis in the CVP. These were *T2R1*, *T2R38*, *T2R40*, and *T2R42*.

Three novel transcripts were identified in this research, potentially encoding new *T2R* receptors: ENSSSCT00000091318.1, ENSSSCT00000089410.2, and ENSSSCT00000017894.3. No meaningful gene names had been previously assigned to these three genes or their encoding transcripts. For the ease of discussion, we assigned the following gene names to them: *T2R61* (encoding ENSSSCT00000089410.2), *T2R62* (encoding ENSSSCT00000091318.1), and *T2R63* (encoding ENSSSCT00000017894.3), as summarized in Table 2. We next investigated the nucleic acid sequence of the three newly identified transcripts, together with the previously reported ENSSSCT00000054601.2 (*T2R20*) by local BLAST search against the pig genome (Sus_scrofa.Scrofa11.1.108). The results showed that with an e-value cutoff of 1 × 10^−30^, the transcript *T2R62* had two hits, while the other three transcripts had one hit (Table 3). None of the hit genes overlapped with any other gene. Three transcripts were from chromosome 5: *T2R62*, *T2R61*, and *T2R20*. Only *T2R63* was from chromosome 18. The two hits of *T2R62*, base pair (bp) 1–776 and bp 777–921, aligned at two regions with 5483 base pairs apart on chromosome 5, compatible with the existence of an intron lying in the middle of the gene encoding for transcript *T2R62* (Table 3). Transcript *T2R61* contained a potential 48 bp long 5′ untranslated region (5′ UTR). The transcripts *T2R20* and *T2R63* contained both a start and a stop codon at the 5′ and 3′ termini, consistent with being mature intronless mRNAs.

### 3.2. PCR Identification of Novel Transcripts

**Figure 2:**
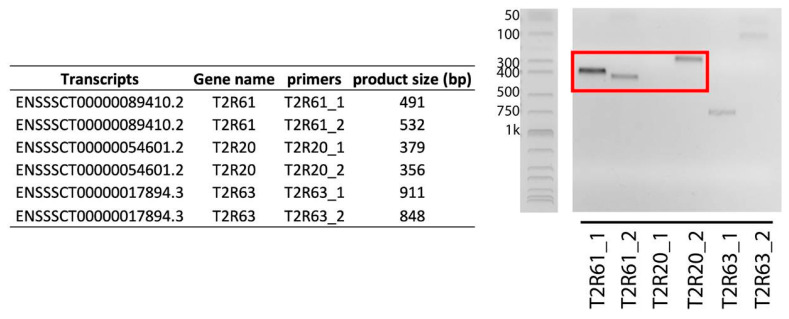
PCR identification of novel transcripts. Sanger sequencing has confirmed three bands (indicated in red) to be consistent with targeted sequence from reference genome.

The PCR results for the intronless transcripts (*T2R61*, *T2R20* and *T2R63*) showed that only *T2R61*, together with *T2R20*, was detectable (Figure 2). The *T2R63* was not identified with positive PCR bands. The subsequent Sanger sequencing confirmed the matching with the targeted area.

**Figure 3:**
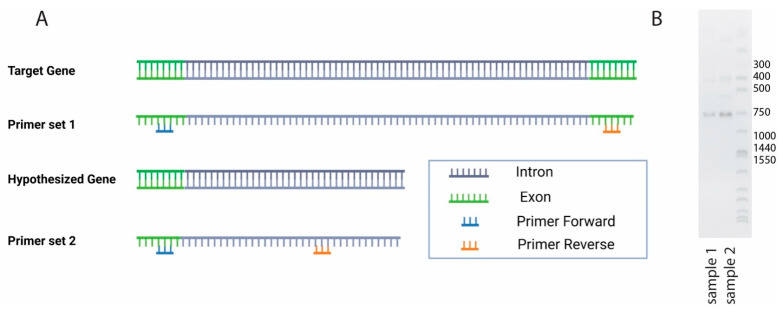
Illustration of hypothesized transcript ENSSSCT00000091318.1 (T2R62) and corresponding primer design (**A**) and PCR identification of primer set 2 against cDNAs from two biological replicates (**B**).

Regarding the *T2R62*, two sets of primers were designed to confirm the expressed transcript (Figure 3A). Primer set 1 (comprising two primer pairs, as in Table 1) was designed to cover exon 1 and exon 2 from the ENSEMBL gene annotation, respectively. Primer set 2 (comprising five primer pairs, as in Table 1) was designed to cover only the sequence with reads from the illumina sequencing result (Figure 3A). The agarose gel showed that one primer pair of primer set 2 (primer 2b) has amplified the target area (Figure 3B). In contrast, primer set 1 did not produce any consistent bands, indicating the absence of the corresponding sequences from the transcriptome.

### 3.3. T2R61 and T2R62 Are Potential Novel T2R Family Members

**Figure 4:**
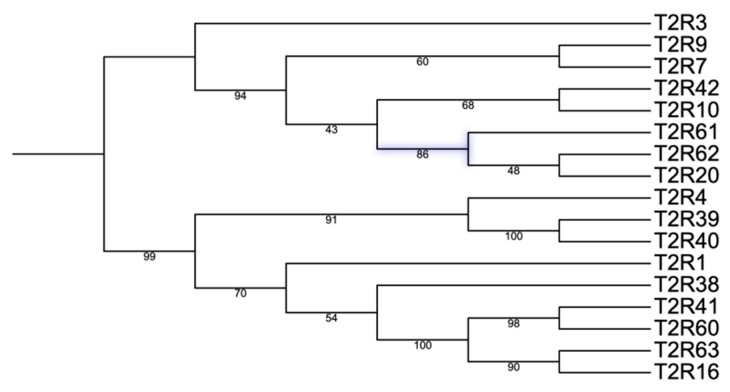
Phylogenetic tree from previously identified pig T2Rs and newly characterized T2Rs (T2R61 and T2R62) in this research with the new branch highlighted in purple.

The gene *T2R62* and its translated protein were both renamed as “*T2R62*” to differentiate from its UniProt recording: A0A5G2QX37 (for more information, the reader is referred to Section 4). The amino acid sequence of all the previously characterized *T2R* transcripts, together with T2R61, T2R62, T2R63 and T2R20 proteins, were subjected to phylogenetic analysis (Figure 4). The phylogenetic trees used gene names for ease of visualization. The phylogenetic tree shows that *T2R20*, *T2R61*, and *T2R62* formed a branch of related sequences. Together, they are close to *T2R3*, *T2R7*, *T2R9*, *T2R10* and *T2R42*.

We next looked at their amino acid sequence homology. The amino acid sequence was deduced from their transcripts by Augustus. Upon comparison of Τ2R61 and T2R62 with the pig proteome using NCBI BLASTp (e-value < 1 × 10^−30^), they showed different levels of identities. The identity percentages for T2R61 with T2R3, T2R7, T2R9, T2R10, and T2R42 ranged from 37.05% to 41.83%. In a similar manner, T2R62 had identity percentages from 34.1% to 37% with the same group of proteins. This was consistent with the phylogenetic tree in Figure 4.

**Figure 5:**
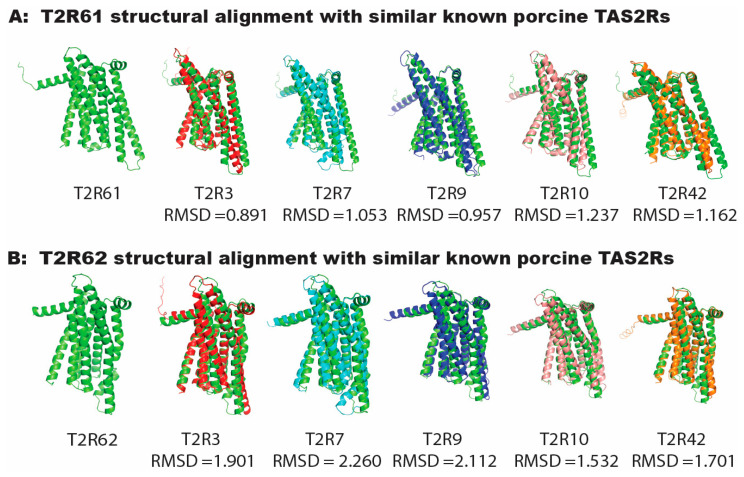
Superimposition of novel transcript encoding protein (green) A0A5G2RA33 (panel (**A**)) and T2R62 (Panel (**B**)) with TAS2R3 (red), TAS2R7 (cyan), TAS2R9 (blue), TAS2R10 (light pink) and TAS2R40 (orange). RMSD = root-mean-square deviation.

A canonical seven-transmembrane domain matching the GPCR and T2R structures was predicted by AlphaFold for both of the proteins (Figure 5A,B, green). A structural superimposition by PyMol was conducted to visualize their structural similarity to T2R3, T2R7, T2R9, T2R10, and T2R42, as measured by root-mean-square deviation (RMSD), which indicated the average distance between the corresponding atoms in two structures after alignment (Figure 5). The RMSD value is generally used to indicate similarity between protein structures. We categorize an RMSD value below 1 Å as good alignment, between 1 Å and 2 Å as moderate good alignment, between 2 Å and 3 Å as acceptable, and above 3 Å as loosely related [33]. The result shows that Τ2Ρ61 (Figure 5A green) has good (RMSD < 1 Å) alignment with T2R3 (Figure 5A red, RMSD = 0.891) and T2R9 (Figure 5A blue, RMSD = 0.957); and moderate good (1 Å < RMSD < 2 Å) alignment with T2R7 (Figure 5A Cyan, RMSD = 1.053), T2R10 (Figure 5A light pink, RMSD = 1.053) and T2R42 (Figure 5A Orange, RMSD = 1.162). For T2R62, it shows moderate good (1 Å < RMSD < 2 Å) alignment with T2R3 (Figure 5B red, RMSD = 1.901), T2R10 (Figure 5B light pink, RMSD = 1.532) and T2R42 (Figure 5B Orange, RMSD = 1.701); and acceptable similarity (2 Å < RMSD < 3 Å) to T2R7 (Figure 5B Cyan, RMSD = 2.260) and T2R9 (Figure 5B blue, RMSD = 2.112).

**Figure 6:**
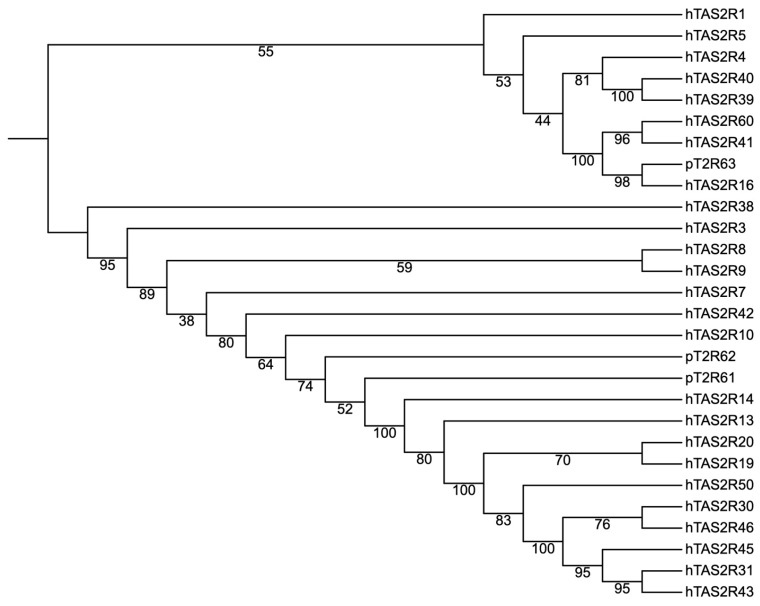
Phylogenetic tree of pig T2R61, T2R62 and T2R63 with known human TAS2Rs.

Due to the limited number of pig T2R studies, we next analyzed the homology of T2R61 and T2R62 with human TAS2Rs to further investigate their phylogenetic relationship. The amino acid sequences of the 25 human bitter taste receptors reported by Meyerhoff and co-workers [14] were aligned together with the three novel pig T2Rs, and a phylogenetic tree was built, as shown in Figure 6. This tree shows that the newly identified novel pig T2R receptors T2R61 and T2R62 are not closely related to any branches of known human TAS2Rs. T2R63 is closely related to human TAS2R16. It is noted that the sequences of the 26th bitter taste receptor TAS2R2 reported by Lang and co-workers [15], were not used in this study.

### 3.4. The T2R20 Protein Is Similar to Multiple Human TAS2Rs

Human TAS2R20 (NCBI Gene ID: 259295, Uniprot Accession P59543, former gene symbol TAS2R49) presented 59.21% identity with the porcine T2R20 protein. However, when the pig T2R20 protein sequence was BLASTed against the human proteome in NCBI, the result showed various degrees of identity to other TAS2Rs as well (e-value < 1 × 10^−30^), with percent identity ranging from 37.04 to 61.43%, including multiple well-known TAS2Rs: TAS2R3, TAS2R7, TAS2R8, TAS2R9, TAS2R10, TAS2R13, TAS2R14, TAS2R42, TAS2R43, TAS2R31 (TAS2R44), TAS2R45, TAS2R46, TAS2R30 (TAS2R47), TAS2R20 (TAS2R49), and TAS2R50.

**Figure 7:**
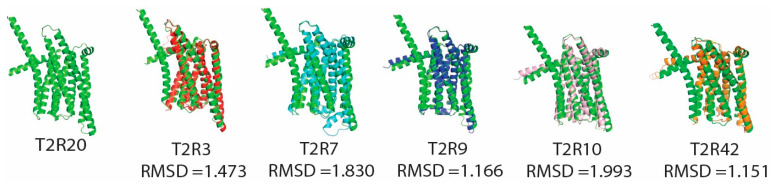
Superimposition of T2R20 protein (green) with T2R3 (red), T2R7 (cyan), T2R9 (blue), T2R10 (light pink), and T2R42 (orange). RMSD = root-mean-square deviation.

In the pig proteome, T2R20 protein showed identities ranging from 35.11 to 40.40%, with previously identified T2R3, T2R7, T2R9, T2R10, and T2R42 at an e-value cutoff of 1 × 10^−30^ which is consistent with the fact that T2R61, T2R62 and T2R20 protein are being placed in the same branch as in Figure 7.

## 4. Discussion

Previously, 15 *T2R* genes (numbers *1*, *3*, *4*, *7*, *9*, *10*, *16*, *20*, *38*, *39*, *40*, *41*, *42*, *60*, *134*) were identified (Table 4) in pigs [4,18]. Among them, the identification of *T2R134* remains questionable, as no complete sequence was reported, and the reference pig genome (Sus_scrofa.Scrofa11.1.108) did not include any annotation about this gene. Therefore, *T2R134* was excluded from further analysis in this study. *T2R38* and *T2R42* transcripts were characterized by PCR or qPCR but were obsolete in the updated Uniprot/Ensembl database. In total, only 12 *T2R* genes with valid previous references were identified in this study. The *T2R20* was annotated as “*taste receptor type 2 member 20-like*” in the pig reference genome (Sus_scrofa.Scrofa11.1.108) and not assigned a Gene ID in NCBI annotation or the Uniprot database. The previous identification of this gene also lacked sequence confirmation. These facts have cast some doubt on the identification/functionality of this gene. One of the outcomes of the analysis that followed in this research was the annotation of this gene as “*T2R20*” and the further verification of its expression.

This study aimed to discover new transcripts that encode for previously unidentified *T2R* genes in pigs. We first directly queried the pig genome using known pig *T2Rs*, and no novel genomic positions were identified. In order to more precisely identify potential pig *T2R* genes, a comprehensive search and analysis of transcriptomic data from 12 pig (*Sus scrofa*) CVP was performed. Quality control by feature summary and PCA analysis showed that the transcriptomic assembly pipeline was of high quality, and no outliers were detected (Figure 1). Multiple transcripts relating to taste bud cell function were characterized, suggesting successful dissection and sample preparation of taste bud tissues. Regarding the identification of novel *T2R* transcripts, three (*T2R61*, *T2R62*, and *T2R63*) were identified with similar deduced amino acid sequences compared to other T2Rs. Two of them were intronless (*T2R61* and *T2R63*), and one (*T2R62*) was annotated in the ENSEMBL database with a 5483 base-pair intervening sequence, possibly indicating the presence of an intron.

For the intronless transcripts *T2R20*, *T2R61* and *T2R63*, we designed primers as shown in Table 1 to amplify the target transcripts and verify their existence. It was verified to cover the transcripts only partially and that the primers were specific. The outcome of the local BLAST of the amplicons against the pig reference genome showed that the hits were in the targeted gene positions, confirming the identity of the genes. The PCR and Sanger sequencing results confirmed the existence of one of the expected novel transcripts: *T2R61* (Figure 2). The *T2R20* expression was also confirmed (Figure 2).

For the *T2R62*, NCBI and ENSEMBL provided different annotations: NCBI recorded only one exon spanning 918 nucleotides, and ENSEMBL recorded two exons with 776 and 142 nucleotides separately. It was noticed that the 5483 bp insertion between the two exons in ENSEMBL annotation would be the first intron in the T2R family reported to date (which was not confirmed). A close inspection of the RNAseq data revealed that only bp 21 to 1798 were covered with reads. In particular, bp 21 to 776 were annotated as exon 1, and bp 777 to 1798 were annotated as part of an intron in the ENSEMBL database. The second exon (bp 6261 to 6405) was not covered with any illumina reads. Based on these observations, we hypothesize that the ENSEMBL annotated intron sequence is incorrect and the actual exon should cover bp 21 to 1798. We used the cDNA of pig CVP tissues as a template to run PCR combined with Sanger sequencing. It is worth noting that our hypothesis tested whether the ENSEMBL annotated “intron” was transcribed into mRNA. Consequently, genomic DNA was used as a template for PCR. This hypothesis was tested by PCR combined with Sanger sequencing. The Sanger sequencing confirmed that the sequence obtained with primer 2b covered the boundary of exon 1 and the putative (now discarded) “intron” of the gene under study. Primers (primer set 1 in Table 1) covering exon 1 and exon 2 did not amplify any consistent band, while primer 2b (Table 1), which covers part of exon 1 and part of “intron”, did amplify, showing a consistent band with the expected nucleic acid sequence. Therefore, it was concluded that the “intron” annotated in ENSEMBLE does not exist. Our result supported that *T2R62* was transcribed from an intronless gene, consistent with other known porcine T2Rs. It is for this reason that *T2R62* translating protein was renamed as “*T2R62*” to avoid confusion with its Uniprot record A0A5G2QX37 in the following analysis. Future work should include verifying the unique intron–exon structure of *T2R62* in other available pig genome assemblies and across different breeds to confirm its conserved genomic architecture.

NCBI annotation has named *T2R62* as “*taste receptor type 2 member 7-like*”, implying it is a duplicate of the pig *T2R7* gene. However, this naming is from automatic computational annotation [37], and its amino acid sequence is very different from other proteins annotated as pig T2R7 in the Uniprot database. Therefore, the NCBI and ENSEMBL annotations about this gene both need updates, and the acronym *T2R62* would be more appropriate to avoid further confusion.

Two of the three potential novel *T2R* transcripts were successfully verified by PCR and Sanger sequencing. The third transcript (*T2R63*) was detected by RNAseq only, but not by PCR. This result could be due to multiple reasons. For example, primer pairs and PCR conditions may need to be further optimized for a successful PCR identification, such as the template amount potentially being too low, amongst other potential reasons. For the following analysis, we focused on the newly identified T2R61 (Uniprot Accession: A0A5G2RA33) and T2R62 (Uniprot Accession: A0A5G2QX37), together with T2R20 (Uniprot Accession: A0A2287A3Q1), for protein sequence and structure characterization. The proteins translated from the two recently discovered transcripts, T2R61 and T2R62, showed different degrees of identity with other known bitter taste receptors (e-value < 1 × 10^−30^): T2R3, T2R7, T2R9, T2R10, and T2R42. The phylogenetic tree (Figure 4) of identified pig T2Rs and novel proteins (T2R61 and T2R62) supported that they may be potential novel T2Rs and not a variant or duplicate of existing known porcine T2Rs. Homology relationships of pig T2R61 and T2R62 with human TAS2Rs remain to be further explored once more comprehensive updates on T2Rs in non-porcine species are available.

In the analysis of protein structures predicted by AlphaFold, T2R61 and T2R62 exhibit a canonical structure with seven transmembrane domains, resembling the GPCR/T2R architecture [38]. Structural superimposition using PyMol was performed to assess their similarity to known receptors (T2R3, T2R7, T2R9, T2R10, and T2R42) based on root-mean-square deviation (RMSD), a measure of structural alignment. T2R61 shows good alignment (RMSD < 1 Å) with T2R3 and T2R9, and moderate alignment (1 Å < RMSD < 2 Å) with T2R7, T2R10, and T2R42. T2R62 displays moderate alignment with T2R3, T2R10, and T2R42, and acceptable alignment with T2R7 and T2R9 (Figure 5).

Overall, the phylogeny (Figure 4) and structural alignment suggested that T2R61 and T2R62 protein structures are similar to some known pig T2Rs. This structural similarity is the basis of T2R61 and T2R62 for potential interaction with bitter compounds, as in other bitter taste receptors. While structural similarity alone cannot determine physiological roles, it provides important foundational information that helps validate the transcript as a plausible member of the porcine T2R family and supports future functional investigations. Therefore, we conclude that *T2R61* and *T2R62* are new gene members of the T2R family in pig.

Although it was attempted to compare the putative ligand binding pocket of T2R61/T2R62 with those of mouse or human T2Rs, this comparison seemed unlikely to result in a prediction of specific agonist due to the fact that pig T2R61 and T2R62 were most closely related to a cluster of primate specific human TAS2Rs (Figure 6) that showed a considerable variance of agonistic molecules, including TAS2R46, TAS2R31, TAS2R43 and TAS2R14, [38,39] according to bitterDB [40].

For the T2R20 protein, the blast result and phylogenetic analysis suggested that the porcine T2R20 protein is not necessarily a one-to-one ortholog of human TAS2R20 but rather forms a clade with multiple human TAS2Rs. The AlphaFold predicted structure, combined with structural alignment by PyMol, showed that the T2R20 protein showed moderately good similarity (1 Å < RMSD < 2 Å) to known pig T2Rs (T2R3, T2R7, T2R9 T2R10, and T2R42), as in Figure 7. It is worth noting that one of the purposes of this research was to see whether the novel proteins convey similar structures to other known T2Rs. The biochemical mechanism of large RMSD value variations and their implications for protein–ligand interaction remains a matter for further research. Our result clarified that the porcine T2R20 protein (A0A287A3Q1) is indeed a member of the porcine T2R family, highly similar to multiple human TAS2Rs, including TAS2R20 (TAS2R49) and many others.

*T2R38* and *T2R42* were not characterized from our RNAseq analysis, and they were removed from the updated Uniprot and Ensembl database, even though their genomic positions were still recorded in NCBI. The exact reason for the removal remains unknown. One possible reason is lacking high-quality data support. Previous PCR and qPCR characterizations of these two transcripts were also lacking robust sequence confirmation. Therefore, the three genes (*T2R38*, *T2R42*, and *T2R134*) were removed from the porcine bitter taste repository. The newest porcine *T2R* gene repository is summarized in Table 4. Here, we propose naming the two genes expressing newly identified T2R transcripts as *T2R61* and *T2R62*. In total, there were 14 porcine T2Rs supported by strong evidence. One T2R transcript (*T2R63*) remains to be further verified. The gene encoding for this potential T2R transcript is proposed to be named *T2R63* (expressing transcripts ENSSSCT00000017894.3 and translating protein F1SSN1).

While the gene models for *T2R61* and *T2R62* partially existed in prediction databases (e.g., UniProt accessions A0A5G2RA33 and A0A5G2QX37), our study provides the first direct experimental evidence of their expression in porcine circumvallate papillae and clarifies the exon structure of *T2R62*, which differs from the existing annotation.

One of the limitations of this research is the lack of functional data on the newly discovered *T2Rs*, which adds to the gap in functional data for other porcine TRs. Nonetheless, transcriptomic and PCR-based characterization provides reliable confirmation of transcript expression, consistent with approaches commonly used in other porcine *T2R* studies. Further investigations are warranted to unravel the taste functions associated with the family of TRs in Sus scrofa. In situ hybridization, qPCR, or RNAscope will be valuable for confirming whether these *T2R* genes are expressed in taste tissues. Another limitation of this study is that it does not address the natural genetic variation (polymorphisms) within the porcine *T2R* repertoire. While the current sample size (*n* = 12) may limit the broader generalizability of these findings across diverse porcine populations, the transcriptomic and PCR-based validations provide a robust framework for this initial characterization. Consequently, this study establishes a necessary foundation for future large-scale verification and functional association studies. Given the role of bitter taste in feed preference and avoidance, the genes identified here, including the novel *T2R61* and *T2R62*, are prime candidates for future association studies. Investigating polymorphisms in these genes and correlating them with production traits such as feed efficiency, growth rate, and diet acceptance in commercial pig populations represents a crucial and logical next step to this work, with significant potential implications for animal nutrition and breeding strategies.

## 5. Conclusions

The pig CVP transcriptomic analysis identified two potentially new or corrected pig *T2R* sequences. One novel transcript (ENSSSCT00000089410.2, named *T2R61*) was confirmed by RNAseq and PCR identification. One transcript ENSSSCT00000091318.1 (named *T2R62*) annotation needs updating, as only one exon is being transcribed, and this exon should at least cover bp 21–1798 in ENSSSCT00000091318.1. The encoded proteins from both transcripts were proposed to be novel T2R family members: T2R61 and T2R62. ENSSSCT00000054601.2 (named *T2R20*) encoded a protein that showed high similarity to multiple human TAS2Rs. The transcript ENSSSCT00000017894.3 (named *T2R63*) was identified by RNAseq but could not be verified by PCR. In total, the porcine *T2R* repository contains 14 transcripts characterized with strong evidence from this and previous research. Moving forward, this work should be extended through functional studies—such as in situ hybridization and qPCR—to confirm cellular expression across multiple tissues. Alternatively, genetic association studies could explore polymorphisms within the *T2R* repertoire to link genetic variation to key production traits (like feed efficiency and diet acceptance), thereby connecting gene discovery to practical applications in pig nutrition and breeding.

## Figures and Tables

**Table 1:** Primers designed for novel transcript identification.

Transcripts	Forward Primer	Reverse Primer	Note	Amplicon Length (bp)
ENSSSCT00000054601	CTGGGAGCTTCGTTCTTCTT	CCTGTTCATTCTGCTGTCTCT		379
ENSSSCT00000054601	CTTGCTACTAGCCTCAGCATATT	GATCCTTTGCCATTGCACTTC		356
ENSSSCT00000089410	GGATCAAGAACAGGAGGTTCTC	AAAGGAGGTCAGGGACACTA		491
ENSSSCT00000089410	CCTGCCTCAGTGTCTTCTATTT	AATGGATGGCCAGATGGATAG		532
ENSSSCT00000091318	CATTCCCTTGAGGCAGGATT	CATTCCCTTGAGGCAGGATT	primer set 1a	846
ENSSSCT00000091318	GCCAACCACTTGAGCATTTG	CATTCCCTTGAGGCAGGATT	primer set 1b	645
ENSSSCT00000091318	ATGGAGGCCCATTTCAGAGC	CCATTTGCTTGGCTGAGAGC	primer set 2a	275
ENSSSCT00000091318	AGTGATTCCAGAGACGCCAG	TCTTTTCAGGCCCCACACTT	primer set 2b	272
ENSSSCT00000091318	GATTCCAGAGACGCCAGCAT	TCACAGGGACTAGGTGGTCATA	primer set 2c	799
ENSSSCT00000091318	AGCATGGAGGCCCATTTCAG	TTCACAGGGACTAGGTGGTCATA	primer set 2d	785
ENSSSCT00000091318	TCTGTGCCGTTCCTGTTCAT	CTGTGAATCCCTGAATGACTGT	primer set 2e	986
ENSSSCT00000017894	TGCCAAGGGAAATCACAGAG	GTGTGGAAGACGAGGAAGAAG		911
ENSSSCT00000017894	GTCTTCCTGGTCGTCTTTGT	CTCTCCTTTAGGGCCTTTCTC		848

Primer set 1 (a,b) was designed to cover exon 1 and exon 2 from the ENSEMBL gene annotation, respectively. Primer set 2 (a to e) was designed to cover only the sequence with reads from the illumina sequencing result on the second exon in transcript ENSSSCT00000091318.

**Table 2:** Transcripts regarded as similar to existing porcine *T2Rs*. The asterisk (*) indicates novel transcripts identified by RNAseq.

Uniprot ID	Ensembl ID	Gene Name
A0A287AYK0	ENSSSCG00000038925.1	*T2R10*
A0A287AA31	ENSSSCG00000037368.1	*T2R16*
I3LF46	ENSSSCG00000028394.2	*T2R3*
I3LCT3	ENSSSCG00000021954.2	*T2R39*
I3LE78	ENSSSCG00000021525.2	*T2R4*
F1SSI6	ENSSSCG00000016457.2	*T2R41*
F1SRW2	ENSSSCG00000016458.3	*T2R60*
A0A286ZVQ9	ENSSSCG00000035471.1	*T2R7*
F1SQ48	ENSSSCG00000000631.2	*T2R9*
A0A287A3Q1	ENSSSCT00000054601.2	*T2R20-like*
A0A5G2QX37 *	ENSSSCT00000091318.1 *	*T2R62* *
A0A5G2RA33 *	ENSSSCT00000089410.2 *	*T2R61* *
F1SSN1 *	ENSSSCT00000017894.3 *	*T2R63* *

**Table 3:** Nucleotide local blast against pig reference genome.

Transcript ID	Query Start	Query End	Subject Start	Subject End	e-Value	Chromosome
ENSSSCT00000091318.1	1	776	61227816	61228591	0	5
ENSSSCT00000091318.1	777	921	61234075	61234219	2.55 × 10^−69^	5
ENSSSCT00000089410.2	25	954	61217359	61218287	0	5
ENSSSCT00000054601.2	1	912	61181330	61182241	0	5
ENSSSCT00000017894.3	1	1176	5568482	5567307	0	18

**Table 4:** Pig *T2R* gene characterization update.

*T2R* Genes	Uniprot Accession	Chromosome	Genomic Coordinates	Previously Characterized?	Characterization Level in This Study
*T2R1*	A0A8W4F831	16	7,573,832–7,574,728	Yes [4]	Not characterized
*T2R3*	I3LF46	18	8,065,985–8,066,932	Yes [34]	RNAseq
*T2R4*	I3LE78	18	8,059,331–8,060,218	Yes [35]	RNAseq
*T2R7*	A0A286ZVQ9	5	154,107–155,042	Yes [4]	RNAseq
*T2R9*	F1SQ48	5	61,253,928–61,254,863	Yes [36]	RNAseq
*T2R10*	A0A287AYK0	5	61,242,636–61,243,565	Yes [36]	RNAseq
*T2R16*	A0A287AA31	18	24,286,430–24,287,329	Yes [36]	RNAseq
*T2R20* *	A0A287A3Q1	5	61,181,330–61,182,241	Yes [4]	RNAseq + PCR
*T2R39*	I3LCT3	18	7,068,087–7,069,214	Yes [4]	RNAseq
*T2R40*	A0A480JCU5	18	7,025,749–7,026,516	Yes [19]	Not characterized
*T2R41*	F1SSI6	18	6,780,808–6,781,731	Yes [4]	RNAseq
*T2R60*	F1SRW2	18	6,806,911–6,808,689	Yes [4]	RNAseq
*T2R61*	A0A5G2RA33	5	61,216,785–61,218,284	No	RNAseq + PCR
*T2R62* *(T2R-new)*	A0A5G2QX37	5	61,227,816–61,234,219	No	RNAseq + PCR
*T2R63*	F1SSN1	5	5,503,228–5,504,400	No	RNAseq
*T2R38* ^#^	Not Applicable	18	7,982,188–7,983,921	Yes [36]	Not characterized
*T2R42* ^#^	Not Applicable	5	61,141,863–61,146,328	Yes [4]	Not characterized
*T2R134* ^#^	Not Applicable	Not Applicable	Not Applicable	No	Not characterized

* Note: *T2R20* was named as “*T2R20-like*” in NCBI annotation. ^#^ *T2R38*, *T2R42* and *T2R134* records were obsolete from Uniprot and Ensembl databases. They were also not characterized by RNAseq analysis in this research. Therefore, they were removed from Porcine Bitter taste repository.

## Data Availability

The resulting data were archived in Sequence Read Archive (SRA) in National Center for Biotechnology Information (NCBI) with BioProject ID: PRJNA1153759.

## Abbreviations

The following abbreviations are used in this manuscript:

T2R	Taste Receptor family 2
TAS2R	Taste Receptor family 2 (Human)
CVP	Circumvallate Papillae
PCA	Principal Component Analysis
PC	Principal Component
UTR	Untranslated Region
GPCR	G protein-coupled receptor
RMSD	Root-Mean-Square Deviation
